# Determinants of clinical, functional and personal recovery for people with schizophrenia and other severe mental illnesses: A cross-sectional analysis

**DOI:** 10.1371/journal.pone.0222378

**Published:** 2019-09-18

**Authors:** Bert-Jan Roosenschoon, Astrid M. Kamperman, Mathijs L. Deen, Jaap van Weeghel, Cornelis L. Mulder

**Affiliations:** 1 ESPRI Epidemiological and Social Psychiatric Research Institute, Department of Psychiatry, Erasmus Medical Center, Rotterdam, the Netherlands; 2 Parnassia Psychiatric Institute, The Hague, the Netherlands; 3 Faculty of Social and Behavioral Sciences, Institute of Psychology, Leiden University, Leiden, the Netherlands; 4 Tilburg University, Department of Social and Behavioral Sciences, TRANZO Scientific Center for Care and Welfare, Tilburg, the Netherlands; 5 Parnassia Psychiatric Institute, Antes/Bavo Europoort, Rotterdam, the Netherlands; Department of Psychiatry and Neuropsychology, Maastricht University Medical Center, NETHERLANDS

## Abstract

**Objective:**

To analyze the relationships between insight, medication adherence, addiction, coping and social support—components of Illness Management and Recovery (IMR)—as determinants of clinical, functional and personal recovery in patients with schizophrenia and other severe mental illnesses. Our rationale lay in the interrelations between these concepts suggested in a conceptual framework of IMR.

**Methods:**

The cross-sectional design used baseline data of outpatient participants in a randomized clinical trial on IMR (N = 187). We used structural equation modeling (SEM) to describe pathways between degrees of insight, medication adherence, addiction, coping and social support, and degree of clinical, functional and personal recovery. We also explored whether clinical recovery mediated functional and personal recovery.

**Results:**

Our final model showed that coping was associated with clinical, functional and personal recovery. Direct associations between coping and functional and personal recovery were stronger than indirect associations via clinical recovery. Although SEM also showed a significant but weak direct pathway between social support and functional recovery, there were no significant pathways either between social support and clinical or personal recovery, or between insight, medication adherence, addiction and any type of recovery.

**Conclusions:**

Coping may be a determinant of all three types of recovery, and social support a determinant of functional recovery. Clinical recovery appears not to be a prerequisite for functional or personal recovery. While our results also suggest the relevance of improving coping skills and of enhancing social support, they only partially support the conceptual framework of IMR.

## Introduction

In recent years the concept of recovery has become increasingly important, and is generating interest and optimism among various stakeholders, such as service users, providers and payers [1]. Treatment success in mental healthcare for people with schizophrenia or other serious and persistent mental illnesses (SMI) is perceived more and more as progress in terms of the degree of recovery. However, recovery is a complex and multidimensional concept, and has been defined in various ways [2–4]. In a typology that is used throughout this paper, three types of recovery can be differentiated, which should not be considered as mutually exclusive, but as complementary aspects of recovery [5]. In this typology recovery is regarded as an outcome and as a cross-sectional reflection of functional status [3]. The first type is clinical or symptomatic recovery, which as defined in the present study, concerns the degree of psychiatric symptomatology [6–8]. This does not equate with symptomatic remission, i.e., the absence or a sustained reduction in symptoms over a certain period and scored below distinct thresholds [2, 7, 9–11].

The second type is functional recovery, also named objective recovery [12]. While some authors equate this with functional remission [7, 9], others consider it to be part of clinical recovery [13–14]. Or they regard cognitive functioning as part of the definition [9, 15–16]. However, in the present study, functional recovery is defined as the degree of vocational and social functioning, such as acting according to age-appropriate role expectations, the performance of daily living tasks without supervision, engagement in social interactions [15], and the degree of independence with regard to housing [4, 9]. Functional recovery thus concerns functional outcomes rather than functional capacity [17–19].

The third type of recovery is personal recovery, a term that originated among people with lived experience of mental illness and also highlights the personal nature of the recovery process [20–21]. Sometimes named subjective recovery [12], it includes components such as spirituality, empowerment, actively accepting the illness, and also finding hope, re-establishing a positive identity, developing meaning in life, overcoming stigma, taking control of one’s own life, and having supporting relationships [7]. In a shorter definition, it concerns the extents of perceived recovery, sense of purpose, and personal agency [12]. To summarize the key elements of personal recovery, various authors use the acronym CHIME: connectedness; hope and optimism about the future; identity; meaning in life; and empowerment [22].

One program for promoting recovery is Illness Management and Recovery (IMR), a curriculum-based psychosocial program for people with SMI that is intended to improve various aspects of illness management and self-management; and also by helping participants to set and achieve personal goals. Its overall purpose is to foster clinical recovery and to support personal and functional recovery. The development of IMR was based on an empirical review of the research literature on teaching illness self-management strategies to people with SMI. In this review, five empirically supported strategies were distinguished: psychoeducation on SMI and its treatment, cognitive-behavioral techniques to medication adherence, developing a relapse prevention plan, enhancing social support by social skills training, and coping skills training for controlling persistent symptoms. Those five illness management strategies were integrated into the IMR program [12, 23]. The theoretical foundation of IMR rests on two models. The first, the trans-theoretical model, holds that people are more motivated to acquire new behavior if the types of intervention are adjusted to the stage of change they are in [24–25]. The second model is the stress-vulnerability model, which holds not only that mental health problems originate from the interaction between biological vulnerability and sources of stress in the environment, but also that people differ in their coping ability [12, 26–27].

The aim of our main study, a randomized clinical trial (RCT) on IMR, is to determine the effectiveness of the IMR program in people with SMI as described in our protocol [28]. The design is to compare the effects of “IMR + Care as Usual (CAU)” with those of “CAU only” on illness management constituents and on the three types of recovery mentioned. Measurement was planned to take place before randomization and at 12 and 18 months after randomization. Generalized linear mixed models (GLMM) are used to investigate group differences between the experimental and control conditions over an 18 months period, including a 12 months treatment period and a 6 months follow-up period.

The working mechanisms of IMR are suggested in a conceptual framework in which better illness management leads to better clinical recovery, and better clinical recovery leads to more personal and functional recovery, see S1 Fig [12, 28]. For the present cross-sectional study we used the data of all 187 people who participated in the baseline measurement of our RCT on IMR. Aim of this analysis is to seek empirical support for any relationships between the concepts suggested in this conceptual framework, but without the input of the IMR-program. We used structural equation modeling (SEM) to develop a recovery-path model on the basis of the direct and indirect associations proposed in this framework. This considers the associations between various components of illness management and the degree of clinical, functional and personal recovery. These components of illness management include psychiatric insight, adherence to medication, substance use, coping and social support, all being important ingredients of the theoretical base of the IMR-training.

We also explored whether clinical recovery does indeed mediate the association between these constituents of illness management and functional and personal recovery. We hypothesized that while the five illness-management constituents had direct pathways to clinical recovery, they had indirect pathways, via clinical recovery, to functional and personal recovery. SEM is a convenient statistical method for exploring such direct and indirect relationships for inferential purposes in a cross-sectional analysis [29–31].

## Methods

### Study participants

In this study baseline data were used of 187 people who agreed to participate in the Dutch RCT on IMR [28]. There were four inclusion criteria for this RCT: (1) having severe and persistent mental illness (SMI), such as a psychotic disorder or a schizoaffective disorder with or without comorbid disorders such as substance abuse and personality disorders; (2) being aged between 18 and 65; (3) currently at or recently admitted to the outpatient mental health services at one of two participating mental healthcare institutions in the Rotterdam area in the Netherlands; and (4) being willing and able to give written informed consent. There were three exclusion criteria: (1) having already participated in IMR training; (2) being unable to give informed consent due to intellectual disabilities; and (3) insufficient knowledge of the Dutch language. Participants in the study were recruited through clinician referrals for IMR from one of the 14 participating community mental health teams.

### Data collection

In interviews with participating clients and their clinicians, research assistants collected information on social-demographic variables: age, gender, living situation, educational level, native country, and source of income, and on psychiatric history: length of treatment and number and length of hospital admissions. This information was later cross-validated in the electronic client files. During the interviews with clinicians, we collected the diagnoses that had previously been made by psychiatrists in the community mental health teams on the basis of clinical interviews: psychotic disorders, mood disorder and/or personality disorder. These were also cross-validated in the electronic client files. In these interviews with clients and clinicians, questionnaires were filled out on illness management, clinical, functional and personal recovery.

This study was conducted in accordance with the Declaration of Helsinki. The RCT was approved by the medical research ethics committee (MREC) at Erasmus MC Rotterdam (Netherlands), and accredited by the Dutch Central Committee on Research Involving Human Subjects (CCMO), and was registered under number NTR 5033 in the Dutch National Trial Register [28].

### Measures

#### Illness management constituents

Coping was assessed using the client-rated Coping Self-Efficacy Scale (CSES). As the 26 items of the CSES are rated on a scale of 0–10, total scores range from 0–260, with higher scores indicating higher self-efficacy for coping with challenges and threats. The CSES consists of three factors: using problem-focused coping (12 items); stopping unpleasant emotions and thoughts (9 items); and getting support from friends and family (5 items) [32]. Social support was assessed using the client-rated Multidimensional Scale of Perceived Social Support (MSPSS), whose 12 items are rated on a scale of 1–7. Total scores therefore range from 12–84, with higher scores indicating higher perceived social support. The MSPSS has a three-subscale structure: family, friends, and significant others [33]. Medication adherence was assessed using the treatment-adherence-subscale of the clinician-rated Service Engagement Scale (SES). As the four items of this subscale are rated on a scale of 0–3, possible total scores of the subscale range from 0–12, with higher scores indicating higher medication adherence. The total SES (14 items) includes four subscales: availability, collaboration, help-seeking, and treatment adherence [34]. Psychiatric insight was measured using the client-rated Insight Scale (IS), which captures three dimensions of insight: perceived need for treatment, awareness of illness, and re-labelling symptoms as pathological [35]. Total scores range from 0–12, with higher scores indicating better insight [36]. Problems with a.) alcohol, or b.) drugs were assessed using one client-rated item (item 24) from the Addiction Severity Index (ASI). As the two sub-items of this item are each rated on a scale of 0–4, possible total scores range from 0–8, with higher scores indicating more substance-related problems. The ASI is a semi-structured interview designed to provide an overall assessment of the severity of problems in seven potential problem areas in substance-abusing patients: medical status, employment and support, drug use, alcohol use, legal status, family/social status, and psychiatric status [37–38].

#### Clinical recovery

Clinical recovery was operationalized as the score on the client-rated Brief Symptom Inventory (BSI), whose 53 items are rated on a scale of 0–4. Total mean scores were used, with higher scores indicating that a client’s symptoms are more severe. The BSI has nine dimensions: psychoticism, depression, somatization, phobic anxiety, obsessive compulsiveness, interpersonal sensitivity, anxiety, hostility, and paranoid ideation [39–41]. To reflect the degree of recovery, the scores were reversed: i.e. a higher total score represents a higher degree of clinical recovery.

#### Functional recovery

Functional recovery was operationalized as the score on the Social Functioning Scale (SFS), a self-administered questionnaire that consists of 76 items with varying response formats. A higher score indicates more, or a higher frequency of, competent behavior. All items are assigned to seven subscales: social engagement/withdrawal; interpersonal behavior; pro-social activities; recreation; independence competence; independence performance; employment/occupation. Each subscale score is the sum of all item values of that subscale, and all subscale values are standardized and normalized to a scaled score (Mean = 100, SD = 15). The full SFS scale score is computed as the mean of the scaled scores of the seven subscales [42–43].

#### Personal recovery

Personal recovery was operationalized as the score on the Mental Health Recovery Measure (MHRM), whose 30 items are rated on a scale of 0–4. Total mean scores were used, with higher scores indicating better personal recovery. The questionnaire consists of three subscales: self-empowerment, learning and new potentials, and spirituality [44–47].

### Statistical analyses

First, Pearson correlations were computed for the five constituents of illness management we measured: insight, coping, social support, medication adherence, and problems with alcohol and drugs; and also for clinical, functional, and personal recovery. Three categories were used in our interpretation of correlations: weak [0.1, 0.3), moderate [0.3, 0.5) and strong [0.5) [48].

To test our recovery-path model, we used SEM. The major advantages of this analysis are the ability to identify direct and indirect pathways and corresponding errors and to examine the associations among multiple independent and dependent variables simultaneously [29].

Our IMR model was tested as follows. First, we fitted the full unconstrained model, i.e. a model including the full set of illness-management constituents (insight, coping, social support, alcohol and drug use, and medication adherence) as independent variables, and functional and personal recovery as dependent variables. Clinical recovery was included as a mediating variable; see S2 Fig. We estimated the size and significance of all direct and indirect paths. Then, in subsequent steps, we removed all paths that did not significantly contribute to the fit of the model. The fit of the final, simplified, model was then tested against the fit of the full model. A non-significant result indicates that the simplified model fits the data as well as the full model does.

To allow for deviation from multivariate normality of the data and missing data-points the fit of the path-model and of the path coefficients were estimated using the robust maximum likelihood estimation-method [49–50]. Chi^2^-tests with Satorra-Bentler correction were used to compare the fit of the nested models [51]. The fit of the final model was evaluated using the following: Chi^2^ and p-value (p>.05) and Chi^2^/df ratio (where a ratio smaller than 1.5 indicates a good fit); the Comparative Fit Index (CFI) [52] and Tucker-Lewis Index (TLI) [53] (where values above .95 indicate a good fit); Root Mean Square Error of Approximation (RMSEA) [54] (where a value lower than.06 indicates a good fit); and Standardized Root Mean Square Residuals (SRMR) [55](where a value lower than.05 indicates a good fit).

As well as using statistical significance of path coefficients, three categories were used in our interpretation of the strength of the relationships: weak [0.2, 0.5), moderate [0.5, 0.8) or strong [0.8) [48]. We used SPSS 23.0 for data-management and descriptive analyses [56]. SEM-analyses were performed using MPlus version 7.4 software [57].

## Results

### Participants’ characteristics

Modal participants were male, living alone, had a secondary educational level, had been born in the Netherlands, and had a psychotic disorder. The modal length of treatment was more than five years, they had been admitted at least three times, had been hospitalized for less than one year, and had an income from unemployment, invalidity or sickness benefit (see Table 1).

**Table 1:** Participants’ characteristics.

		*N*	%
total		187	100%
gender	male	99	53%
	female	88	47%
living situation			
alone		111	59%
with partner/family		48	26%
in institution^1^		28	15%
education level			
primary		69	37%
secondary		79	42%
higher		39	21%
native country			
Dutch		136	72%
Western immigrant		16	9%
Non-western immigrant		35	19%
source of income			
employment		12	7%
benefit for unemployment, invalidity/sickness benefit		126	67%
social security benefit		41	22%
no income		6	3%
missing		2	1%
diagnosis^2^			
psychotic disorders		106	57%
mood disorder		61	33%
personality disorder		58	35%
length of treatment			
≤ 5 years		47	25%
> 5 years		139	74%
missing		1	1%
number of admissions			
None		48	26%
1–2		69	37%
≥ 3		70	37%
length of hospitalization			
not hospitalized		48	26%
≤ 1 year		94	50%
> 1 year		45	24%
		*M*	*SD*
Age (years)		44.29	10.38

^1^ sheltered living or in hospital

^2^ one person can have had more than one diagnosis

### Correlations

Table 2 presents the correlation coefficients of the constituents of illness management, and of degrees of clinical, functional, and personal recovery. Significant correlations are highlighted. The correlation of functional and personal recovery was strong. Clinical recovery had a moderate correlation with functional recovery, and a strong correlation with personal recovery. Functional recovery had a strong correlation with coping, a moderate correlation with social support, a weak negative correlation with medication adherence, and a weak correlation with alcohol and drug use. Personal recovery had a strong correlation with coping, a moderate correlation with social support, and a weak negative correlation with insight and medication adherence.

**Table 2:** Correlations (and standard errors), mean scores, and standard deviations for constituents of illness management, and for degrees of clinical, functional and personal recovery.

	Functional	Personal	Clinical	Coping	Social	Insight	Medication	Alcohol and
	Recovery	Recovery	Recovery		Support		Adherence	Drug Use
Functional Recovery	1.00							
Personal Recovery	0.56 (0.06)***	1.00						
Clinical Recovery	0.44 (0.07)***	0.60 (0.06)***	1.00					
Coping	0.58 (0.06)***	0.75 (0.05)***	0.61 (0.06)***	1.00				
Social Support	0.36 (0.07)***	0.37 (0.07)***	0.19 (0.07)**	0.42 (0.07)***	1.00			
Insight	-0.11 (0.07)	-0.25 (0.07)**	-0.18 (0.07)*	-0.34 (0.07)***	-0.03 (.07)	1.00		
Medication Adherence	-0.22 (0.07)**	-0.16 (0.07)*	-0.11 (0.07)	-0.25 (0.07)**	-0.05 (.07)	0.14 (.07)	1.00	
Alcohol and Drug Use	0.15 (0.07)*	0.00 (0.07)	0.04 (0.07)	0.09 (0.7)	0.15 (.07)*	0.13 (.07)	-0.05 (0.07)	1.00
M	105.54	69.70	1.26	134.74	4.90	9.65	10.78	0.47
SD	8.66	20.19	0.85	50.75	1.51	2.88	1.90	1.18

* p<0.05

** p<0.01

*** p<0.001

### Structural equation modeling

In accordance with the paths we hypothesized, our exploration of the effects consisted of three pathways: 1. exploration of the direct pathways between illness-management constituents (coping, social support, psychiatric insight, addiction, treatment adherence) and clinical recovery; 2. exploration of the direct pathways between clinical recovery and functional and personal recovery; and 3. exploration of the indirect pathways that lie, via clinical recovery, between the constituents of illness management and functional and personal recovery.

**Fig 1:**
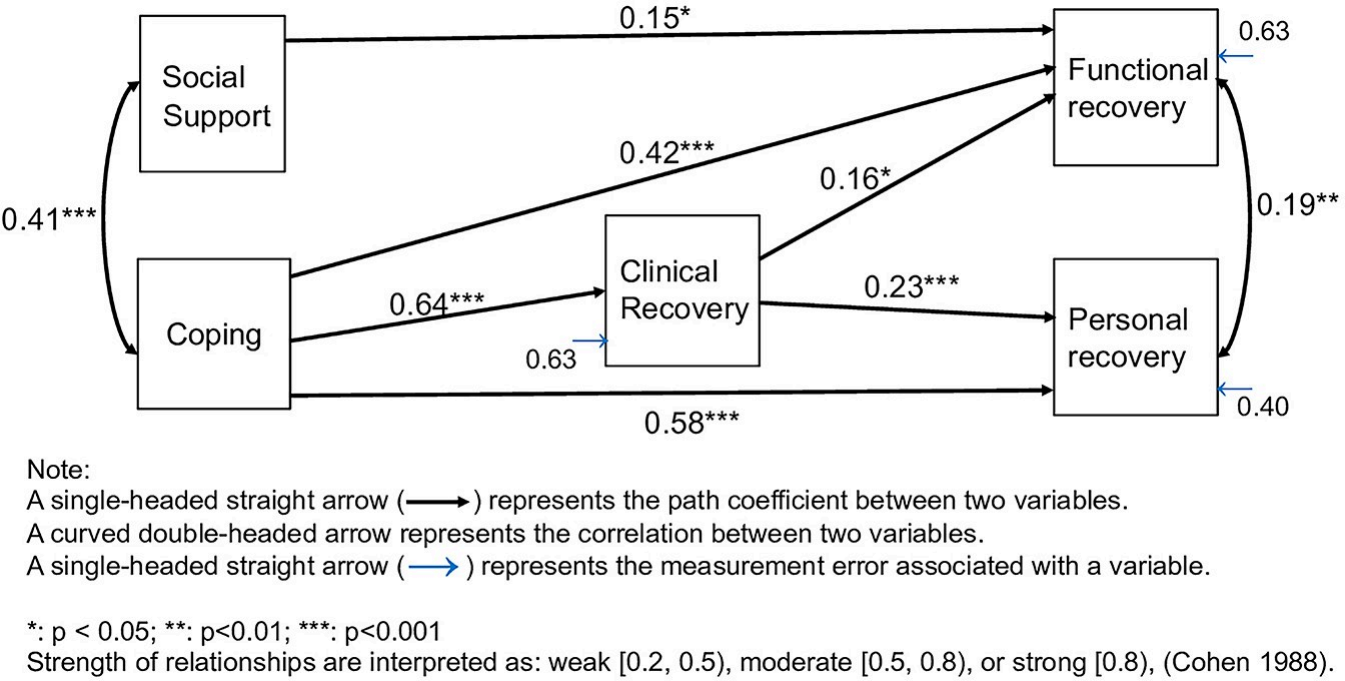
Final model showing significant standardized path coefficients of illness management constituents, and clinical, functional, and personal recovery for people with SMI.

First, we fitted a full, unconstrained model, i.e., a model containing all the paths between the full set of the specified illness-management components and the recovery variables. A simplified model was then constructed by subsequently removing all paths that did not significantly contribute to the fit of the model. The final model fitted the data well: Chi^2^ = 13.96; df = 11; Chi^2^/df = 1.27; p>0.05; CFI = 0.99; TLI = 0.98; RMSEA = 0.038 (90%CI = 0.000–0.090); SRMR = 0.028. This simplified model consisted of paths between coping, social support, clinical recovery, and functional and personal recovery. Table 3 shows all the direct and indirect path coefficients of the simplified model. Significant paths are shown in Fig 1.

**Table 3:** Final model: Standardized direct and indirect path coefficients and standard errors for the pathways between coping, social support, clinical recovery and functional and personal recovery for people with SMI.

	Functional Recovery		Personal Recovery	
	Estimate	SE	Estimate	SE
Coping				
*Direct path*	0.418***	0.077	0.579***	0.074
*Indirect paths*				
Coping- Clinical Recovery-Functional Recovery and Personal Recovery	0.103*	0.049	0.146**	0.042
Total	0.521***	0.059	0.725***	0.051
Social Support				
*Direct path*	0.151*	0.063	0.083	0.064
*Indirect paths*				
Social Support- Clinical Recovery- Functional Recovery and Personal Recovery	-0.012	0.012	-0.016	0.017
Total	0.139*	0.063	0.067	0.066
Clinical Recovery				
*Direct path*	0.161*	0.073	0.229***	0.065

* p <0.05

** p<0.01

*** p<0.001

Coping was highly significantly, directly and moderately associated with the degree of clinical and personal recovery. Coping was highly significantly, directly and weakly associated with the degree of functional recovery. Via clinical recovery, coping was found to have significant indirect pathways to functional and personal recovery. However the direct associations between coping and functional and personal recovery were much stronger than the indirect associations.

The SEM showed a minor significant direct pathway between social support and functional recovery; the small path coefficient suggested a weak association. There were no significant direct or indirect paths between social support and clinical or personal recovery. This was also the case between insight, medication adherence, addiction and any type of recovery; but this may have been because scores in these domains showed limited variance.

## Discussion

### Main findings

This study was cross-sectional in design, with baseline data for 187 clients from an RCT used to examine pathways between specific IMR components—insight, medication adherence, addiction, coping and social support—and degree of clinical, functional and personal recovery, utilizing SEM. We also sought to explore whether clinical recovery mediated functional and personal recovery. By using baseline data we did not seek to examine the impact of the IMR program but empirical support for the associations between the concepts proposed by the conceptual framework of IMR.

We found that coping was directly and strongly associated with personal and functional recovery; we also found that these associations were moderated only marginally by clinical recovery. Therefore, coping seems to be more important for the degree of functional and personal recovery than the level of clinical recovery is. We found a weak direct pathway between social support and functional recovery. Insight, medication adherence, alcohol and drug use were not associated with any type of recovery.

As Fig 1 shows, we found partial empirical support for the conceptual framework of IMR–see S1 Fig—(1) there was a strong, direct pathway between coping–a component of illness management—and the degree of clinical recovery; and (2) clinical recovery was associated, albeit marginally, with functional and personal recovery. However, in a divergence from the conceptual framework, there were strong, direct associations between coping and the degree of functional and personal recovery. Social support—also a component of illness management—was not associated with clinical recovery, but had a weak direct association with functional recovery. In another divergence from the conceptual framework, there were no relationships between the three other constituents of illness management we measured and the degree of clinical recovery [12]. And, in a third divergence from the conceptual framework, clinical recovery appeared not to be a prerequisite for personal and functional recovery.

Our finding that clinical recovery was not a prerequisite for functional recovery is in line with the results of earlier studies that reported that functional recovery had occurred in certain patients despite continuing symptoms [58–60] and with another study that concluded that no sequential relationship could be suggested between symptomatic remission and functional remission [61]. In line with our finding of a significant but weak pathway between clinical recovery and personal recovery, a recent meta-analysis showed that the association between clinical and personal recovery is small to medium, and that personal recovery was explained only partly by symptom severity. The same meta-analysis concluded that treatment and outcome monitoring of patients with schizophrenia spectrum disorders should pay attention separately to clinical and personal recovery [8].

Our results underscore the clinical relevance of interventions aiming to improve coping skills as a means of promoting progress in clinical, functional, and personal recovery, such as those in the IMR-modules “Coping with stress” and “Coping with problems and persistent symptoms”. And our findings also confirm the relevance of interventions that focus on improving social support in order to promote progress in functional recovery, the IMR-module “Building social support” being one such intervention.

### Strengths/Limitations

The strengths of the study include a large sample size, a broad range of measures–including those addressing the components of recovery–and analyses that comprise a more comprehensive understanding of predictors of recovery.

As, in their view, recovery models and frameworks are founded mainly on qualitative studies and expert opinion, Slade et al. have indicated an evidence gap [5]. Such a gap might be bridged by our study, which is based on the conceptual framework of IMR, uses quantitative data from standardized recovery measures, and aims explicitly to explore the determinants of clinical, functional and personal recovery.

And although most of our study population consisted of people with psychotic disorders, our inclusion of patients with other serious mental illnesses contributes to generalizability in this domain, and also to external validity.

Our study also has various limitations. First, although coping and personal recovery are different theoretical concepts, they may have some overlap. However, the content of most of the CSES items is different from that of the MHRM items, especially regarding the items of the two largest subscales of the CSES, (1) Use problem-focused coping and (2) Stop unpleasant emotions and thoughts. Secondly: due to our use of specific definitions and operationalization’s of the three differentiated types of recovery, the generalizability of our results is limited in concepts of recovery that have been defined otherwise.

The third limitation is our inability to find significant pathways between addiction, medication adherence or insight and the degree of clinical, personal and functional recovery. This may have lain in the fact that a majority of the participants in our study had few problems in these domains and therefore showed only limited variance on the scores.

Fourth, as our analysis was cross-sectional and not longitudinal, we were unable to determine temporal causality. And as our analysis was led by the directions of the associations suggested in the conceptual frame work of IMR, we did not explore reverse directions of the associations between the various concepts.

### Potential future directions

We have chosen to use total scores of most measures. More specific results could be obtained, if analyses were repeated using subscales of the illness management constituents and of the MHRM, but also using other components of personal recovery, such as self-esteem or self-stigma. Secondly: the generalizability of this type of research on recovery could increase if there would be more uniformity in definitions and operationalization’s of the different types of recovery—see introduction. Thirdly: to gain more insight into the impact of addiction, medication adherence and insight on the different types of recovery a research population could be chosen having more variance on the scores on these domains. Fourth: to be able to determine temporal causality this study could be repeated with a longitudinal analysis.“

## Conclusions

Our results suggest that coping is a determinant of all three types of recovery, and that social support is a determinant of functional recovery. Clinical recovery appears not to be a prerequisite for functional and personal recovery. Our results also suggest the relevance of improving coping skills for clinical, functional and personal recovery, and of fostering social support for functional recovery. The conceptual framework of IMR could be only partially supported.

## Supporting information

S1 Fig Conceptual framework for Illness Management and Recovery [12]. (TIF)

S2 Fig Full model including all hypothesized paths between illness management components, and clinical, functional, and personal recovery for people with psychotic disorders and other serious mental illnesses. (TIF)
