# Supplementary figures and images for: Determinants of clinical, functional and personal recovery for people with schizophrenia and other severe mental illnesses: A cross-sectional analysis
Source: PLoS One. 2019 Sep 18;14(9):e0222378. doi: 10.1371/journal.pone.0222378 (PMC6750648; doi:10.1371/journal.pone.0222378)

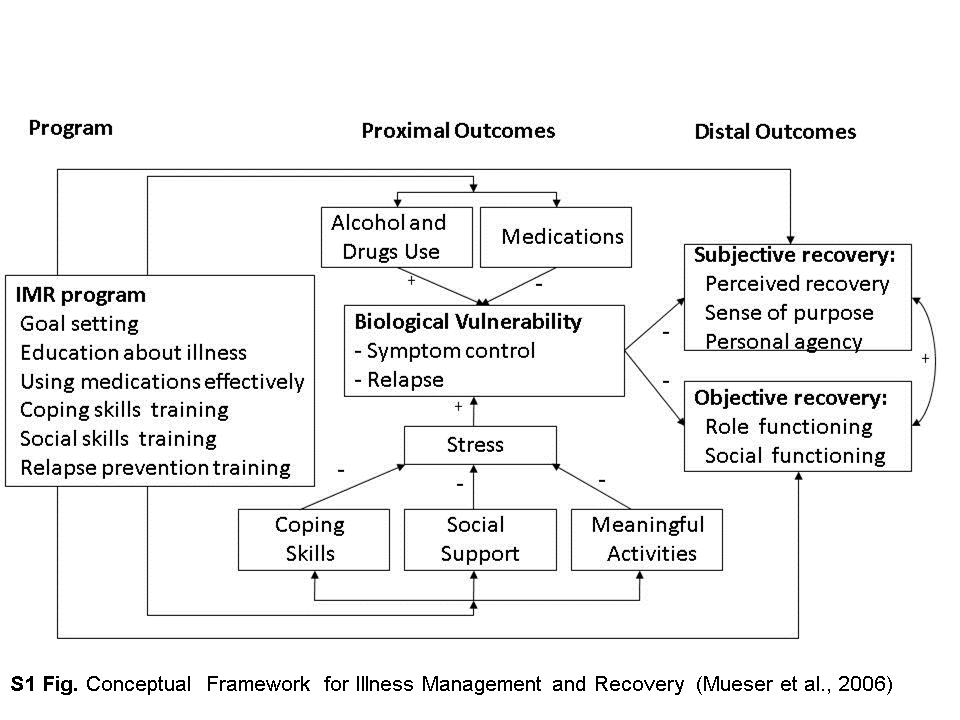

Supplement: S1 Fig — (TIF) [file pone.0222378.s001.tif]

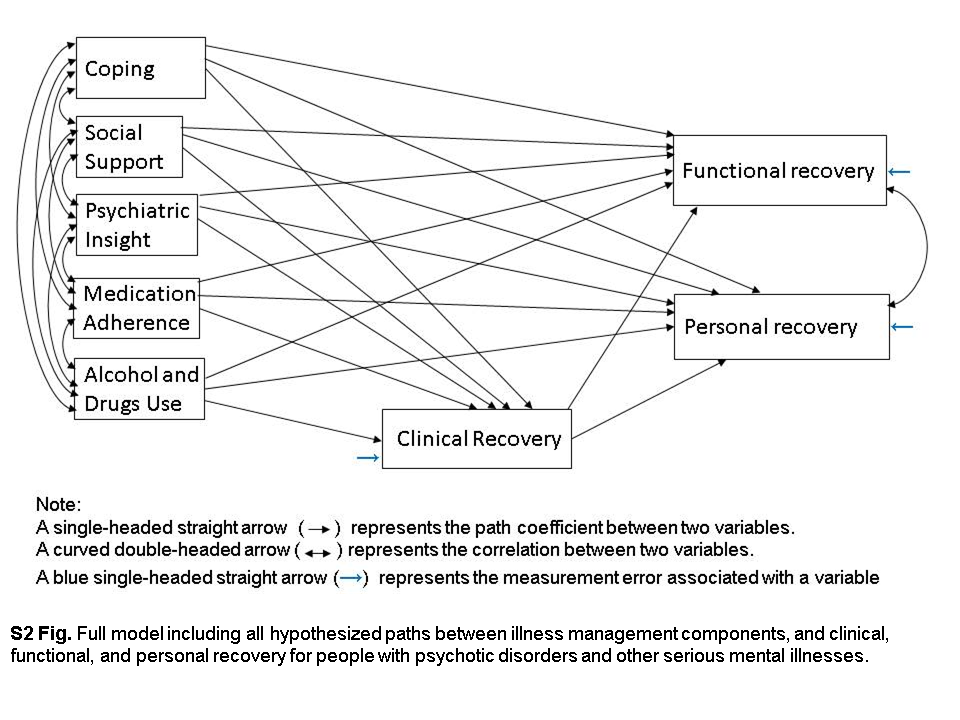

Supplement: S2 Fig — (TIF) [file pone.0222378.s002.tif]
